# The epidemiology and deprivation profile of firearm-related injuries and deaths in British Columbia, Canada

**DOI:** 10.24095/hpcdp.45.6.03

**Published:** 2025-06

**Authors:** Mojgan Karbakhsh, Fahra Rajabali, Alex Zheng, Ian Pike

**Affiliations:** 1 BC Injury Research and Prevention Unit, BC Children’s Hospital Research Institute, Vancouver, British Columbia, Canada; 2 Department of Pediatrics, The University of British Columbia, Vancouver, British Columbia, Canada

**Keywords:** firearms, Canada, socioeconomic factors, rural, suicide, injuries, gunshot, mortality

## Abstract

**Introduction:**

Firearm-related injuries (FRI) are an important public health issue in Canada. This study aims to determine the incidence of FRI in British Columbia (BC) and examine the distribution according to demographics, intent, urban–rural residence and neighbourhood deprivation.

**Methods:**

De-identified data on deaths and hospitalizations (2010–2019) were retrieved from the BC Vital Statistics and the Discharge Abstract Database obtained from the BC Ministry of Health. We implemented the Canadian Index of Multiple Deprivation for the dissemination area–level marginalization.

**Results:**

A total of 1868 fatal and nonfatal FRI were included in our study, of which 46.4% were due to self-harm. The annual injury rate was 3.93 per 100000, with the highest rates among men aged 15 to 34 years. Rates were highest in rural and remote areas, in neighbourhoods with the least diverse ethno-cultural composition, and the greatest level of situational vulnerability and economic dependency. We did not observe significantly different rates across residential instability quintiles. The marginalization pattern for intentional self-harm was similar to the aggregated deprivation profile. While assaults were more common in neighbourhoods with higher levels of situational vulnerability and more diverse populations, unintentional injuries were more prevalent in neighbourhoods with higher levels of situational vulnerability.

**Conclusion:**

This study revealed that the burden of FRI was not evenly distributed across demographic determinants, neighbourhood deprivation or urban–rural areas of residence throughout BC. We also observed different deprivation profiles across the various intents of injury and death. Findings highlight the need for addressing FRI at its root causes, by implementing system-level interventions focussed on suicide prevention, poverty reduction, and promoting employment and education.

**Highlights**

- Between 2010 and 2019, a total of 1035 British Columbians lost their lives and another 833 were seriously injured due to firearms.
- Intentional self-harm made up 46.4% of all firearm injuries and deaths in British Columbia.
- The highest rates were among men aged 15 to 34 years, and individuals in rural and remote areas of British Columbia.
- Neighbourhoods with less diverse populations, greater situational vulnerability and higher economic dependency had higher rates of serious or fatal firearm injuries.
- Findings highlight the need for addressing firearm-related injuries at the root causes, through suicide prevention and poverty reduction, and promoting employment and education.

## Introduction

Firearm-related injuries (FRI) are a significant public health issue in Canada. According to the Global Burden of Disease study, an estimated 875 fatal FRI occurred among Canadians in 2019, with the death rate being more than three times higher than rates in Australia, England and Ireland.1 With a disability-adjusted life years (DALYs) rate of 108.9 per 100 000 in 2019, FRI were responsible for 39 789 DALYs among Canadians (3854 years of life lost and 35 935 years lived with disability).^1^

Suicide accounts for more than three-quarters of all firearm-related deaths in Canada, more than double the global rate of 0.68 per 100 000 population.^1^ When comparing firearm-related suicide rates in high-income countries with populations greater than 10 million, Canada ranks third, after the US and Chile.^2^ At the same time, a Canadian study demonstrated that assault and unintentional injuries were the most common external causes of nonfatal firearm hospitalizations and emergency department (ED) visits, respectively.^3^

The rate of firearm-related violent crime in Canada began an upward trend in 2014, resulting in an increase of 20% in the six-year period from 2015 to 2020 compared to the previous six years. In 2017, an increase in homicides in two provinces (British Columbia [BC] and Quebec [QC]) was responsible for the national increase in homicides. The increase in BC occurred in both urban and rural areas, which is partly explained by increased numbers of gang- and firearm-related homicides.^4^ The rise in firearm-related homicides in BC was due to increases in Vancouver (+7 victims), Abbotsford-Mission (+4) and non-CMAs* (+10); the increase in QC was the result of the January 2017 mass shooting at a Qubec City mosque (+6).^4^

In 2020, notable increases in rates of firearm-related violent crime were observed in some Canadian jurisdictions, including southern rural BC, where the rate increased by 34%.^6^ The estimated annual cost of violent firearm crime in BC amounts to $294.4 million, with human costs (including health care, productivity and value of statistical life) making up 64% of the total, followed by criminal justice system and programming costs.^7^

While there are estimates that some 26% of Canadian households own at least one firearm, the distribution varies considerably across provinces and territories.^8^ According to the International Crime Victim Survey conducted in 1996, the majority of Canadian households that owned firearms possessed at least one long gun; while BC residents reported owning handguns more often than other Canadians (16% of gun owners).^9^ Another study on the regional variations in methods of self-protection by Canadians between 1999 and 2004 revealed that British Columbians were two times more likely to own guns for protection and 10% more likely to carry weapons compared to residents of Ontario (ON).^10^ That study also demonstrated that Canadians living in rural areas were 2.3 times more likely to own guns, but had lower levels of weapon carrying, suggesting that living in rural areas might be influenced by a culture of firearm ownership used for hunting, protection against wild animals, self-defense against criminals and for entertainment. 10

Previous studies have demonstrated that neighbourhood deprivation is one of the key determinants of the incidence and severity of injuries, including FRI. For instance, a population-based study in ON demonstrated that young men living in the lowest-income urban neighbourhoods were overrepresented in nonfatal assault FRI.^11^ A study of the neighbourhood socioeconomic status of serious injuries in Greater Vancouver, BC, demonstrated higher rates of severe injury in areas with high social and material deprivation, with social deprivation explaining slightly more variation in the injury rates than material deprivation.^12^ The urban–rural divide has also been recognized as a determinant of the rate, type and outcome of FRI—due to the differences in the availability of firearms and access to advanced prehospital and hospital trauma care.^13^,^14^

Despite the notable burden and costs, the epidemiology of FRI and the distribution across deprivation levels and the urban–rural spectrum have not been thoroughly examined in BC. Knowledge of the determinants and patterns of FRI are essential to shape policies and practices in an effort to decrease the burden. Furthermore, much of the epidemiological literature on FRI draws on US studies. Thus, providing evidence on the Canadian context would be beneficial for prevention and policy making. The purpose of this study is to estimate the incidence rate of firearm-related serious injuries and deaths among residents of BC, and to examine the rates across the neighbourhood deprivation spectrum and urban–rural area of residence. The objectives of this study are to determine (1) the crude and standardized rates of FRI in BC; (2) the temporal trend overall and according to intent across the study period; and (3) the distribution of FRI according to sex, age group, intent, firearm type, urban–rural area of residence and neighbourhood deprivation.

*A CMA (census metropolitan area) is an area consisting or one of more adjacent neighbouring municipalities around a large urban area known as the urban core. A CMA must have a total population of at least 100 000 of which 50 000 or more live in the urban core. A non-CMA refers to a geographical region that does not meet the criteria of a CMA.^5^

## Methods

***Ethics approval***

Ethics approval was granted by The University of British Columbia’s Children’s and Women’s Research Ethics Board, #H22-03453.

***Data source and analysis***

Following ethics approval, de-identified data on firearm-related deaths of BC residents between the 2010 and 2019 calendar years were retrieved from the BC Vital Statistics obtained from the BC Ministry of Health, through a data sharing agreement with the BC Injury Research and Prevention Unit (BCIRPU; data pulled Aug 2022). De-identified firearm-related hospitalization data were obtained from the Discharge Abstract Database (DAD) obtained from the BC Ministry of Health, retrieved through the BCIRPU, and the records pertaining to in-hospital deaths (n=51) were removed to avoid the double-counting of fatalities. DAD is a national database, managed by the Canadian Institute for Health Information (CIHI), which contains administrative, demographic and clinical information on hospital separations (also called discharges) from all Canadian provinces and territories, except QC.

The main outcome variable in this study was a fatal or nonfatal injury secondary to firearms during the study period, as identified using the 10th revision of the International Statistical Classification of Diseases and Related Health Problems, Canadian Modification (ICD-10-CA) codes, and examined as counts, proportions and rates per 100000 population. The explanatory variables included sex, age group, intent (assault, intentional self-harm, unintentional, legal intervention and undetermined), firearm type (rifle, shotgun and larger firearm; handgun; BB gun; airgun; other specified; and unspecified firearm), neighbourhood deprivation quintile and urban–rural area of residence. 

We used the Canadian Index of Multiple Deprivation (CIMD), which can be used as a proxy for individual-level deprivation and marginalization.^15^ This geography-based index has been developed by Statistics Canada based on 2016 census microdata at the dissemination area (DA) level, the smallest standard geographical area for which all census data are disseminated. CIMD allows for an in-depth understanding of area-level inequality using quintiles across four dimensions: ethno-cultural composition, situational vulnerability, economic dependency and residential instability. For each of the dimensions, the first quintile represents the least deprived area and the fifth quintile represents the most deprived. For the ethno-cultural composition, quintiles 1 and 5 represent neighbourhoods with the least and most diverse population, respectively. In addition to the national level, three provincial and two regional CIMD indexes are available.^15^ The BC-specific index was linked to the administrative data, using DA as the common identifier. 

Firearm-related injury incidence rates per 100 000 population across the 10-year study period were calculated by summing the total number of fatal and nonfatal injuries and dividing it by the BC population for the same time period. Age- and sex-standardized, population-based, annual incidence rate of firearm-related injuries in BC were calculated using the 2016 Census of Population as the standard population, to enable comparison with other provinces. Ninety-five percent confidence intervals (CIs) for rates were developed at each quintile across dimensions of the CIMD using the Wilson score interval.^16^ Simple linear regression was implemented to investigate the temporal trends overall, and according to intent. 

The urban–rural area of residence was classified according to the seven-tier Community Health Service Area (CHSA) urban–rural categories (metropolitan, large urban, medium urban, small urban, rural hub, rural and remote), based on the 2016 census.^17^ Health services in BC are delivered within five administrative health boundaries (known as health authorities), made up of 89 local health areas. Nested within local health areas, there are 218 CHSAs and 7208 DAs. For the purpose of this study, the DA of the area of residence was used to identify the CHSA urban–rural category, and injury rates were calculated for each category. Frequencies for rural and remote areas were further aggregated to facilitate comparison, as similar rates were observed in these two categories. 

Case fatality rate (CFR) was calculated as the proportion of fatal injuries due to firearms, divided by the total number of firearm-related injuries, stratified by the main study variables and reported as percentages. 

Data analysis was conducted using SPSS Statistics version 26 (IBM Corp., Armonk, NY, US) and the chi-square test was used to examine differences between the distribution of intent and urban–rural area as well as between the CFR and urban–rural area, with the significance level of α≤0.05.

## Results

A total of 1868 BC residents sustained firearm-related injuries during the study period (55.4% fatal), an annual incidence rate of 3.93 per 100 000 and an age- and sex-standardized rate of 3.90 per 100 000 population. The annual death rate by firearms was 2.18, with a standardized rate of 2.11 per 100000 population. 

The mean (SD) age in years of included individuals was 43.4 (19.3); 34.7(14.1) among nonfatal and 50.0(20.0) among fatal cases. Injuries from intentional self-harm accounted for 46.4% of FRI, followed by assault injuries (29.2%) and unintentional injuries (19.1%). Distribution of the frequency and rates of firearm injuries according to demographics, intent and urban–rural area of residence is provided in Table 1. The majority of injured individuals were male (91.8%). Females accounted for 13.0% of assaults and 13.6% of injuries with undetermined intent (vs. 9.3% of unintentional and 4.6% of intentional self-harm injuries). 

**Table 1:** Frequency and rates of firearm injuries according to demographics, intent and urban–rural area of residence, British Columbia, 2010 to 2019

Characteristic	Categories	n (%)	Rate^a^ (95% CI)	CFR (%)
Sex	Male	1714 (91.8)	7.28 (6.95–7.63)	56.0
	Female	154 (8.2)	0.64 (0.55–0.75)	48.7
Age group (years)	Under 15	23 (1.2)	0.33 (0.22–0.50)	21.7
	15–24	338 (18.1)	5.69 (5.11–6.33)	37.9
	25–34	410 (21.9)	6.20 (5.63–6.83)	37.6
	35–44	267 (14.3)	4.24 (3.76–4.78)	44.6
	45–54	275 (14.7)	3.91 (3.47–4.39)	57.5
	55–64	245 (13.1)	3.68 (3.25–4.17)	77.6
	65–74	162 (8.7)	3.57 (3.06–4.16)	87.0
	75–84	105 (5.6)	4.34 (3.59–5.25)	95.2
	≥ 85	43 (2.3)	4.11 (3.05–5.54)	93.0
Intent	Assault	545 (29.2)	1.15 (1.06–1.35)	36.1
	Intentional self-harm	867 (46.4)	1.83 (1.71–1.95)	92.7
	Unintentional	356 (19.1)	0.75 (0.68–0.83)	4.8
	Legal intervention	41 (2.2)	0.09 (0.06–1.17)	24.4
	Undetermined	59 (3.2)	0.12 (0.09–1.60)	11.9
Urban–rural	Metropolitan	590 (31.6)	2.59 (2.39–2.80)	41.2
	Large urban	162 (8.7)	2.55 (2.19–2.97)	51.9
	Medium urban	352 (18.8)	5.44 (4.90–6.04)	52.6
	Small urban	168 (9.0)	5.07 (4.36–5.89)	61.3
	Rural hub	107 (5.7)	4.86 (4.02–5.87)	69.2
	Rural	398 (21.3)	8.01 (7.27–8.84)	72.4
	Remote	27 (1.4)	7.84 (5.39–10.79)	74.1
	Missing	64 (3.4)	NA	59.4

**Data source:** BC Vital Statistics and the Discharge Abstract Database obtained from the BC Ministry of Health.

**Abbreviations:** BC, British Columbia; CFR, case fatality rate; NA, not available.

**Note:** Percentages represent relative frequencies in column; N = 1868.

^a^ Rate per 100 000. 

**Figure 1:**
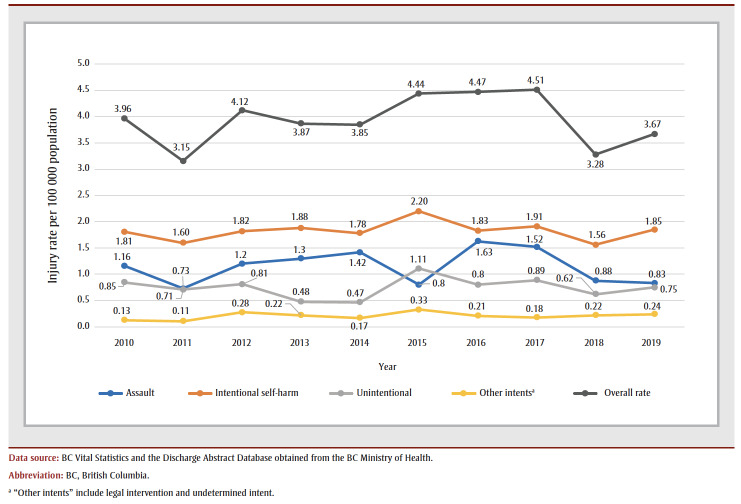
Rates of firearm-related injuries by year and intent, British Columbia, 2010 to 2019

There were no significant temporal trends in rates over the course of the study period, either overall or according to intent (Figure 1).

**Figure 2:**
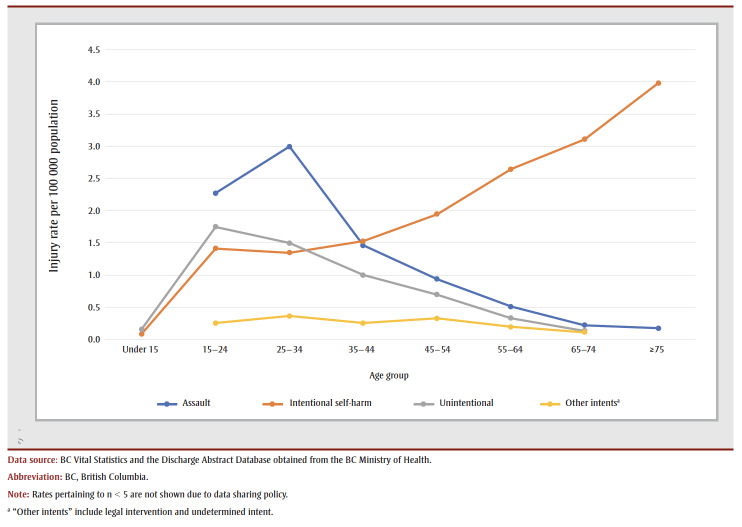
Rates of firearm-related injuries by age group and intent, British Columbia, 2010 to 2019

The highest rates of FRI (including all intents) were among those aged 15 to 24 and 25 to 34 years: 5.69 (95% CI: 5.11–6.33) and 6.20 (95% CI: 5.63–6.83), respectively (Table 1). The highest rates of unintentional injury and assault FRI were observed in these two age groups, while individuals aged 75 years and older had the greatest intentional self-harm rates by firearms (Figure 2). The highest age- and sex-specific FRI rates were observed among men aged 15 to 24 and 25 to 34 years: 11.22 (9.77–12.29) and 11.16 (10.08–12.36) per 100 000 population, respectively (data not shown).

The highest rates of FRI were observed in rural and remote areas (8.00 per 100000, 95% CI: 7.44–9.00), accounting for 22.7% of FRI. The highest CFR was also observed in rural and remote areas, where 72.5% of FRIs were fatal, followed by 69.2% in rural hub; the lowest CFR was 41.2% in metropolitan areas (χ^2^=110.8; *p*<0.001; *df*=5; Table 1).

Assaults and unintentional injuries were overrepresented in metropolitan areas (50.1% and 41.9%, respectively), while 34.3% of firearm-related intentional self-harm occurred in rural and remote areas (χ^2^=236.9; *p*<0.001; *df* = 20; Table 2).

**Table 2:** Demographic characteristics and urban–rural area of residence of firearm-related injuries stratified by intent, British Columbia, 2010 to 2019

Characteristic	Categories	Intent							
		Assault		Intentional self-harm		Unintentional		Undetermined	
		n (%)	CFR (%)	n (%)	CFR (%)	n (%)	CFR (%)	n (%)	CFR (%)
Sex	Male	474 (87.0)	33.5	827 (95.4)	93.2	33 (9.3)	4.6	51 (86.4)	9.8
	Female	71 (13.0)	53.5	40 (4.6)	82.5	323 (90.7)	6.1	8 (13.6)	25.0
Age group (years)	Under 15	NS	NS	6 (0.7)	66.7	11 (3.1)	9.1	NS	NS
	15–24	135 (24.8)	34.8	84 (9.7)	85.7	104 (29.2)	6.7	12 (20.3)	8.3
	25–34	198 (36.3)	34.8	89 (10.3)	91.0	99 (27.8)	1.0	17 (28.8)	17.6
	35–44	92 (16.9)	32.6	96 (11.1)	92.7	63 (17.7)	0.0	6 (10.2)	0.0
	45–54	66 (12.1)	37.9	137 (15.8)	89.1	49 (13.8)	4.1	6 (10.2)	0.0
	55–64	34 (6.2)	44.1	176 (20.3)	96.6	22 (6.2)	9.1	13 (22)	23.1
	≥ 65	16 (2.9)	68.8	279 (32.2)	95.3	8 (2.2)	50.0	NS	NS
Urban–rural^a^	Metropolitan	266 (50.1)	36.1	146 (17.5)	95.2	145 (41.9)	1.4	14 (25.0)	7.1
	Large urban	42 (7.9)	35.7	76 (9.1)	88.2	36 (10.4)	2.8	NS	NS
	Medium urban	110 (20.7)	32.7	160 (19.2)	90.0	66 (19.1)	3.0	11 (19.6)	9.1
	Small urban	29 (5.5)	24.1	102 (12.2)	93.1	27 (7.8)	0.0	8 (14.3)	0.0
	Rural hub	20 (3.8)	45.0	64 (7.7)	96.9	17 (4.9)	11.8	5 (8.9)	20.0
	Rural and remote	64 (12.1)	42.2	286 (34.3)	93.4	55 (15.9)	18.2	15 (26.8)	20.0

**Data source:** BC Vital Statistics and the Discharge Abstract Database obtained from the BC Ministry of Health.

**Abbreviations: **BC, British Columbia; CFR, case fatality rate; NS: not shown due to data sharing policy for counts less than 5.

**Note:** The cases with legal intervention as the intent are not included in this table, due to small cell counts after stratification; percentages represent relative frequencies within each column.

^a^ The urban–rural data were missing for 64 individuals (3.4%) and the proportions are valid percentages among the cases not missing data. 

Almost 21% of FRI were caused by rifles, shotguns or larger firearms; 10.9% by handguns; and 2.4% by nonpowdered guns (i.e. BB and airguns). The remainder were caused by “other” firearms (25.5%) or the type of firearm was missing (40.7%). Rifles, shotguns or larger firearms were involved in more than a quarter of intentional self-harm cases, but in fewer instances of unintentional injuries and assaults (17.7% and 12.7%, respectively). Examining the firearm type across the urban–rural area of residence showed that FRI from rifles, shotguns or larger firearms were more common in small urban areas (33.9%), followed by rural hub (29.9%) and rural and remote areas (28.2%). In contrast, FRI from handguns were more frequent in large urban areas (15.4%), followed by metropolitan areas (12.7%). Stratifying by both intent and urban–rural area of residence further demonstrated that rifles, shotguns or larger firearms were involved in 36.3% of intentional self-harm cases that occurred in small urban areas, in 35.3% of unintentional FRI in rural hubs and 30.0% of assaults in rural hubs.

A nonhomogenous distribution of FRI across the four dimensions of CIMD was observed, in which rates were greatest in neighbourhoods with the least diverse ethno-cultural composition and in neighbourhoods with the most deprived situational vulnerability and economic dependency. Rates were not significantly different across quintiles of residential instability. Stratification by intent further revealed that the disparity in rates among the ethno-cultural composition quintiles was mainly driven by differences in rates of intentional self-harm, although higher FRI rates in the fifth quintile of situational vulnerability were observed for intentional self-harm, assault and unintentional injuries (Figure 3, A–D). 

**Figure 3:**
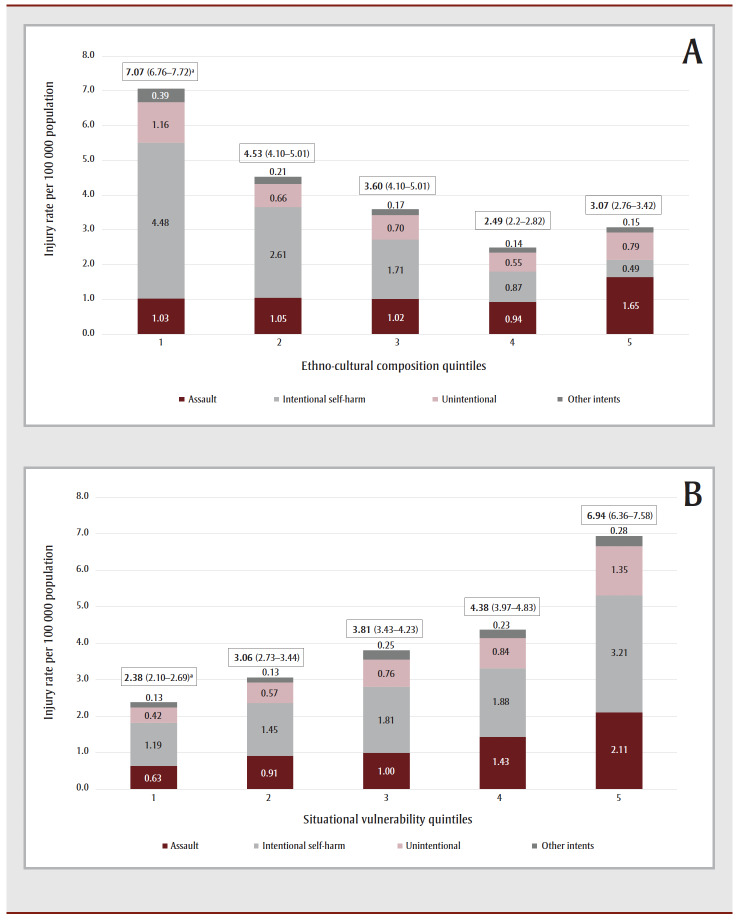
Rates of firearm-related injuries by neighbourhood deprivation quintiles stratified by intent, British Columbia, 2010 to 2019

**Figure 3:**
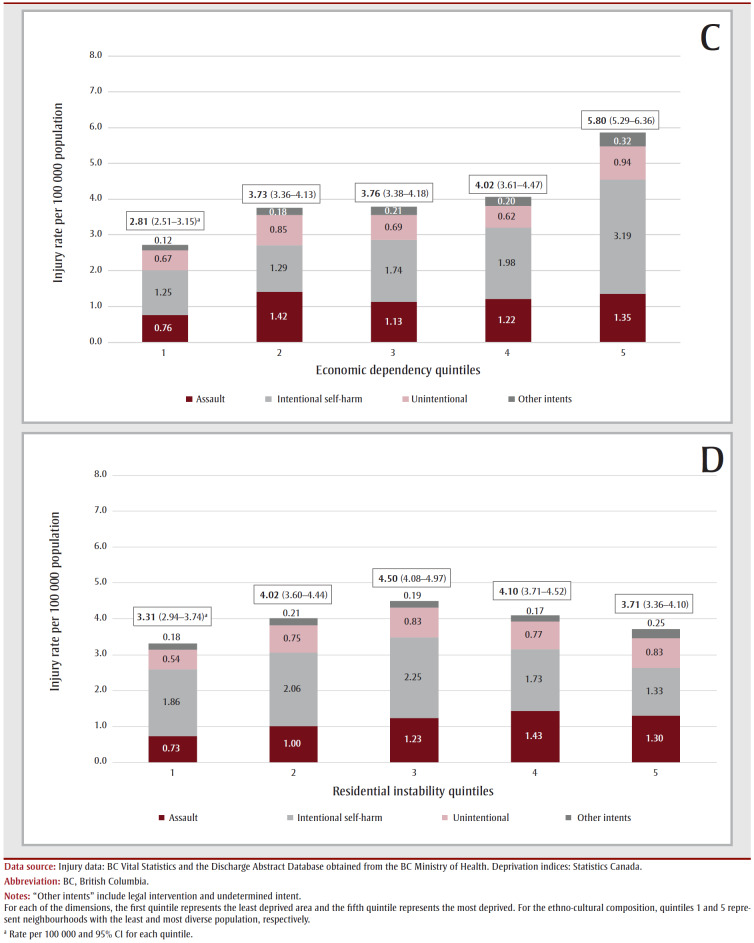
Rates of firearm-related injuries by neighbourhood deprivation quintiles stratified by intent, British Columbia, 2010 to 2019

FRI due to intentional self-harm were more common in neighbourhoods with less diverse populations (37.5% in quintile 1 of ethno-cultural composition), and in higher levels of economic dependency and situational vulnerability (30.0% and 27.8% in quintile 5 of the corresponding dimensions, respectively). Assault-related FRI were predominant in neighbourhoods with more diverse populations (34.1% in quintile 5), and in higher levels of situational vulnerability (28.8% in quintile 5). Unintentional FRI were more common in neighbourhoods with higher levels of situational vulnerability (28.4% in quintile 5; Table 3).

**Table 3:** Distribution of firearm-related injuries and case fatality rate according to quintiles of deprivation dimensions and intent, British Columbia, 2010 to 2019

Deprivation dimension	Q	n (%)^a^	CFR (%)	Intent							
				Assault		Intentional self-harm		Unintentional		Undetermined	
				%	CFR (%)	%	CFR (%)	%	CFR (%)	%	CFR (%)
Ethno-cultural composition	1	492 (27.4)	67.1	13.6	37.5	37.5	92.6	23.7	11.1	35.2	10.5
	2	385 (21.5)	64.4	16.9	42.7	26.7	91.9	16.4	7.1	27.8	13.3
	3	330 (18.4)	52.7	17.6	24.7	18.9	92.4	18.7	1.6	16.7	11.1
	4	250 (13.9)	50.8	17.8	44.7	10.5	93.1	16.1	3.6	11.1	16.7
	5	336 (18.7)	34.5	34.1	33.3	6.5	98.1	25.1	1.2	9.3	0.0
Situational vulnerability	1	248 (13.8)	63.3	12.5	53.0	14.9	97.6	12.9	0.0	11.1	0.0
	2	296 (16.5)	54.7	16.7	34.1	16.8	90.0	16.1	7.3	18.5	20.0
	3	348 (19.4)	56.6	17.2	34.1	19.8	95.8	20.2	4.3	20.4	9.1
	4	401 (22.4)	52.1	24.8	33.6	20.7	90.7	22.5	5.2	22.2	16.7
	5	500 (27.9)	54.0	28.8	32.9	27.8	91.3	28.4	6.2	27.8	6.7
Economic dependency	1	296 (16.5)	53.7	15.2	38.8	15.9	92.4	20.8	5.6	9.3	20.0
	2	361 (20.1)	47.6	25.9	32.1	15.0	98.4	24.0	1.2	18.5	10.0
	3	344 (19.2)	54.7	19.5	39.8	19.1	89.9	18.4	3.2	24.1	0.0
	4	337 (18.8)	58.5	19.3	32.4	20.0	94.6	15.2	5.8	18.5	10.0
	5	455 (25.4)	61.3	20.1	38.7	30.0	90.8	21.6	9.5	29.6	18.0
Residential instability	1	263 (14.7)	62.4	11.0	32.8	17.8	95.3	12.6	0.0	14.8	12.5
	2	353 (19.7)	60.9	16.7	44.3	21.8	93.4	19.3	6.1	16.7	11.1
	3	398 (22.2)	59.0	20.6	39.4	23.9	92.0	21.3	8.2	24.1	15.4
	4	389 (21.7)	54.5	25.8	40.4	19.7	91.5	21.3	4.1	20.4	18.2
	5	390 (21.8)	43.3	25.9	24.8	16.8	92.1	25.4	4.6	24.1	0.0

**Data sources:** Injury data: BC Vital Statistics and the Discharge Abstract Database obtained from the BC Ministry of Health. Deprivation indices: Statistics Canada.

**Abbreviations:** BC, British Columbia; CFR, case fatality rate; Q, quintile. 

**Notes:** The cases with legal intervention as the intent are not demonstrated in this table, due to small cell counts after stratification. Percentages represent relative frequencies within each column. 

^a^ The deprivation quintile was missing for 75 individuals (4.0%) and the proportions are valid percentages among the non-missing cases. 

## Discussion

This study revealed that the burden of serious firearm injuries and deaths was not evenly distributed across demographic determinants, neighbourhood deprivation and urban–rural areas of residence throughout BC. The annual rate of FRI leading to death or hospitalization in BC between 2010 and 2019 was 3.93 per 100 000 population (age- and sex-adjusted rate: 3.90 per 100 000 population), which was similar to the standardized rate in ON (3.54 per 100 000 population), but lower than in Nova Scotia (NS; 4.44 per 100000 population).^11^,^13^ Toigo et al.^3^ examined FRI rates in Canada using three administrative databases capturing deaths, hospitalizations and ED visits and found significant differences across provinces and territories, which can be partially attributed to urban–rural differences in firearm ownership. According to these researchers, the rates of firearm-related death and hospitalization in Canada—excluding hospitalizations in QC—were 2.13 and 2.22 per 100000 population, respectively, for the study period (2016–2020). More than three-quarters of firearm-related hospitalizations occurred in the three provinces of ON, Alberta (AB) and BC, while two-thirds of firearm-related deaths occurred in ON, QC and AB.^3^

Consistent with previous reports, the rate of FRI was highest in 2017 (4.51 per 100 000).^4^ Nevertheless, no significant trends were detected across the study period, either overall or stratified by intent. The overall CFR in our study was 55.4%; however, CFRs were higher in rural and remote areas and higher in older age groups. The overall CFR in this study was higher than that reported by Gomez et al. in ON, and may be related to the lower proportion of self-harm injuries in their study compared to the BC setting.^11^ Another reason for the lower CFR in Gomez et al. might be that the investigators also included FRI visits at ED, lowering the aggregate injury severities.^11^ In comparison, Karkada et al. reported major trauma patients in NS who sustained FRI, with a CFR of 72%, where 64.9% of the FRI were related to intentional self-harm.^13^

British Columbians over the age of 75years had the highest rate of firearm-related self-harm injuries (3.98 per 100 000 population). The Canadian literature has highlighted that the proportion of firearm-related suicide deaths (of all suicide deaths) significantly increases with age.^18^,^19^ Several factors may contribute to this, including a greater proportion of older adults living in rural and remote areas of BC, bereavement of a close person, mental and physical health conditions and financial constraints, and thus, targeted interventions for older adults are warranted while using comprehensive approaches.^20^,^21^

This study showed that the highest rate of FRI occurred in rural and remote areas, mainly driven by suicide among men aged over 45 years. Burrows et al. observed that the risk of death by firearm suicides was 3.4 times higher in rural and remote areas of Canada compared to very large urban areas, while such a difference was not detected for other measures of suicide.^22^ Being male and living in rural areas were among the significant risk factors associated with suicide deaths among older adults in ON, as shown in another study utilizing linked health care administrative databases.^23^ While a literature review has suggested access to firearms, socioeconomic status, limited access to mental health services, stigma and social isolation as other contributing factors to higher firearm-related suicide mortality among older males in rural areas,^24^ additional research is required to examine this disparity across the life course. 

In our study, the information on the firearm type was not available in a considerable proportion of records; however, rifles, shotguns or larger firearms were noted to be involved in 20.6% and handguns in 10.9% of FRI. Finley et al., who examined the contributing factors of firearm-related in-hospital mortality in Canadian trauma centres, also reported that the firearm type was missing in around 50% of cases.^25^ Similar to other Canadian studies, rifles, shotguns or larger firearms were more common in intentional self-harm injuries and among residents of small urban, rural hub and rural and remote areas. In contrast, injuries related to handguns were more prevalent in large urban and metropolitan areas.^26^

We observed higher FRI rates among individuals residing in neighbourhoods within the first quintile of ethno-cultural composition (i.e. areas with lower proportions of individuals who self-identify as a visible minority,† are foreign-born, have no knowledge of either official language or are recent immigrants). A population-based study of residents of ON aged 24 years and younger demonstrated that nonimmigrants had higher rates of unintentional FRI compared to immigrants; however, the rate of assault-related firearm injuries was similar.^27^ While there is a need for further exploration of injury rates and patterns in immigrant populations in Canada, cultural factors and living in urban centres with high-density immigrant communities may be protective against the risk of experiencing some types of injuries.^28^

This study showed higher rates of FRI among individuals living in greater situational vulnerability. A study of the association between suicide and homicide rates in the presence of firearm availability in Canadian provinces (1981–2016) concluded that firearms legislation had no beneficial effect on the overall rates, while higher unemployment, low income and higher proportion of Indigenous population were directly associated with rates of firearm-related suicide.^29^ The rates of FRI were also higher in neighbourhoods with greater economic dependency. This was similar to the findings of Gomez et al., who observed higher rates of FRI in areas of ON with the lowest neighbourhood income.^11^

We did not observe significantly different rates across quintiles of residential instability. The results of previous studies of the association of community-level residential instability and FRI are mixed.^30^,^31^ Areas with higher levels of residential instability may represent populations who face challenges in maintaining their place of residence and thus are susceptible to shifting relationships with the community, and are exposed to fluctuating levels of formal and informal supports.^32^ Further studies are required to examine the association of this neighbourhood-level deprivation dimension with FRI in the Canadian context. 

A novel finding of this study was different deprivation profiles across differing intents of injury and death. The pattern of marginalization for self-harm FRI cases was similar to the overall pattern (i.e. overrepresentation in areas with a less diverse population and with higher economic and situational vulnerability). This was expected, as 46.4% of the FRI were due to intentional self-harm. While unintentional injuries were more prevalent in neighbourhoods with higher levels of situational vulnerability, assaults were more common in neighbourhoods with more diverse populations as well as areas with higher levels of situational vulnerability. While small sample size after stratification by five deprivation quintiles and by intent did not allow statistical comparison within the strata, further studies are required to examine how the neighbourhood deprivation patterns might differ across intents. 

As the CIMD has not been previously implemented to examine disparities in the context of FRI, there is limited evidence with which to compare our findings; however, Saunders et al. observed lower or similar rates of assaults among immigrant youths compared to nonimmigrants in ON, except for firearm assaults, and except for interpersonal injuries by cutting or piercing.^27^

The federal legislative and regulatory actions in Canada include the tightening of rules for obtaining firearms licenses and registration; limiting personal ownership of handguns as well as the ability to buy, sell and transfer them within the country; implementing background checks for firearm purchases and taking away firearms licenses for those involved in domestic violence or criminal harassment; and mandatory firearms safety courses for first-time license applicants.^33^,^34^

While public opinion polls have historically demonstrated strong support of Canadians for tougher gun laws, the *Ending the Long-Gun Registry Act* was a piece of legislation that generated much controversy, augmented by the increasing costs of licensing and registration. Ultimately, this act instituted a major policy change in 2012, eliminating the long-gun registry and requiring registration only for restricted firearms.^35^,^36^ Some scholars believe that the *Common Sense Firearms Licensing Act* further watered down several aspects of the Canadian gun control policies in 2015, by loosening restrictions on the transportation of ﬁrearms within the province of possession. This act also drew criticism from pro-gun groups, as it required a classroom-based ﬁrearms safety course for ﬁrst-time licensees; the groups argued that this resulted in undue hardship for rural and northern residents.^37^,^38^

The research to date evaluating the effectiveness of Canada’s gun control legislation has produced mixed results, depending on the study period and outcomes of interest.^10^,^18^,^39^ While the multiplicity of contributing factors underlying FRI complicates the ability to identify any single effective intervention, policy makers might need to accept this degree of uncertainty and consider implementing packages of legal measures that are more likely to result in major benefits.^40^ Evidence from 130 studies across 10 jurisdictions suggests that in certain countries the simultaneous implementation of laws targeting multiple elements of firearms restrictions has been associated with a decreased number of firearm-related deaths.^41^ While further investigation is required to explore the impact of legislation on FRI in Canada, there is a need for research and public education on the safe storage practices. A survey on the storage of household firearms in QC conducted in 1994 demonstrated that 35% of respondents who kept long guns in their homes had failed to comply with Canadian firearm storage regulations.^42^ While the evidence supports the benefits of safe firearm storage practices, having a gun in the home has shown to be associated with an increased risk of suicide as well as firearm homicide in the household.^43^,^44^

The study findings highlight the need for addressing FRI at its root causes, by implementing system-level changes to reduce disparities. The intersectionality of material deprivation with FRI calls for examining the problem with a public health lens and adopting strength-based solutions focussed on poverty reduction, housing, employment and education.^11^,^45^

While assault incidents in metropolitan areas are highlighted by the media, the burden of FRI in BC is mainly driven by suicide deaths in rural and remote areas. This emphasizes the importance of implementing evidence-based suicide prevention programs, including education on the safe storage of firearms, and promoting social connectedness and support, especially among older adult males, who might be otherwise neglected in the preventive initiatives.

***Strengths and limitations***

While our study is among the first to report the epidemiology of FRI in BC, it has some limitations, particularly that the results are based on existing data drawn from administrative databases. The estimation of the rates was based on hospitalization and death records, and did not include ED visits. The National Ambulatory Care Reporting System (NACRS) is a tool for collecting data and reporting all levels of ambulatory care including ED visits in Canada. In BC, only 30 hospitals report ED data to the NACRS, and it excludes the external cause of injuries—needed for delineating FRI—which is not reported in the available provincial ED data.^46^

Furthermore, the higher proportion of suicide deaths noted in this study might be partially due to not including ED visits (without hospitalization). Cases of intentional self-harm are usually more severe and would require hospitalization beyond the ED, whereas unintentional injuries may be sufficiently treated solely in an ED setting. This lack of consistency in the collection and availability of specific data within the province and across Canada could have an impact on the reported FRI rates and comparability among provinces. 

Another limitation pertains to a lack of sufficient data necessary to parse out the FRI due to interpersonal violence (IPV). While some studies have explored the critical intersection of firearms access and IPV,^47^ we were not able to delineate cases of IPV from among all FRI related to assault. Future research is essential to examine the impact of Canadian legislation on gender-based violence. This is especially the case for the more recent act entitled *An Act to amend certain Acts and to make certain consequential amendments (firearms)*, which received Royal Assent in December 2023 and allows for an emergency prohibition order in order to remove firearms from individuals who may be a danger to themselves or others. 

In addition, some IPV survivors—especially in rural-remote areas—might not seek medical care after sustaining firearm injuries due to the fear of legal consequences and stigma.^48^,^49^ While further studies are required to examine this in the Canadian context, it is less likely to have impacted our results due to the scope of our study including only hospitalizations and deaths.

A further limitation was the implementation of the neighbourhood multiple deprivation index as a proxy for individual-level information. The dissemination area–based index has the potential for ecological fallacy, since not everyone who lives in an area identified as deprived is necessarily affected or marginalized in the same way. Nevertheless, in the absence of comprehensive individual-level information, this index is valuable, providing a profile of the population and potentially facilitating public health action, and this approach is generally accepted in the research literature.^15^

In our setting, information on the urban–rural area of residence was missing for 3.4% of individuals, and the overall proportion of missing information for determination of deprivation quintiles was 4.0%. This missing proportion is considered small and is not expected to affect any interpretation of results.^50^

An additional limitation of the study was the lack of availability of recent data, specifically, for 2020 to the present. The coroner investigation processes can take several months before a final cause of death is assigned, and additional time might be required to update vital statistics registries. The social and economic circumstances around the COVID-19 pandemic and the postpandemic social and economic circumstances may have affected real and reported FRI rates and patterns, and further studies are needed to examine any impact. Despite this limitation, this study provides a baseline for the ongoing surveillance and policy making, as well as for examining the impact of public health interventions on the burden of FRI in the province. 

†Terminology used in the CIMD definition.

## Conclusion

Between 2010 and 2019, a total of 1035 British Columbians lost their lives, and another 833 were seriously injured, due to firearms. The highest rates of FRI were observed among men aged 15 to 24 and 25 to 34 years; among residents of rural and remote areas; and in neighbourhoods with less diverse populations and greater situational vulnerability and economic dependency. 

The association between neighbourhood deprivation and FRI highlights the need for targeted interventions in overrepresented populations. A multifaceted, collaborative approach by policy makers, public health professionals and health care providers is required to reduce poverty and systemic inequalities, and to implement evidence-based suicide prevention initiatives. While progress is being made to understand the incidence, determinants, patterns and impacts of FRI across Canada and in other countries, much more needs to be done to move toward a safer societal approach to gun control, to address the underlying disparities and to effectively reduce the burden of FRI, especially among the overrepresented populations. 

## Acknowledgements

A part of this research was accepted as an oral presentation at SAFETY 2024, the 15th World Conference on Injury Prevention and Safety Promotion.

## Funding

The authors of this study did not receive any specific grants from funding agencies in the public, commercial or not-for-profit sectors.

## Conflicts of interest

The authors declare that they have no competing interests.

## Authors’ contributions and statement

MK: conceptualization, methodology, data curation, formal analysis, writing—original draft, writing—review and editing.

FR: conceptualization, methodology, data curation, writing—review and editing.

AZ: methodology, data curation, formal analysis, writing—review and editing.

IP: conceptualization, supervision, writing—review and editing.

All authors have read and agreed to the published version of the manuscript.

The content and views expressed in this article are those of the authors and do not necessarily reflect those of the Government of Canada.

## References

[B01] Global Burden of Disease Study 2019 Results [Internet]. IHME.

[B02] On gun violence, the United States is an outlier. IMHE.

[B03] Toigo S, Pollock NJ, Liu L, Contreras G, McFaull SR, Thompson W (2023). Fatal and non-fatal firearm-related injuries in Canada, 2016–2020: a population-based study using three administrative databases. Inj Epidemiol.

[B04] Homicide in Canada, 2017. Statistics Canada.

[B05] Dictionary, Census of Population, 2021: census metropolitan area (CMA) and census agglomeration (CA). Statistics Canada.

[B06] Allen M (2022). Trends in firearm-related violent crime in Canada, 2009 to 2020. Trends in firearm-related violent crime in Canada, 2009 to 2020. Juristat.

[B07] Rajabali F, Turcotte K, Zheng A, et al (2022). The cost of firearm violent crime in British Columbia, Canada. Front Public Health.

[B08] Dandurand Y Firearms, accidental deaths, suicides and violent crime: an updated review of the literature with special reference to the Canadian situation [Internet]. Department of Justice Canada.

[B09] Block R Firearms in Canada and eight other western countries: selected findings of the 1996 international crime (victim) survey. Canadian Firearms Centre, Department of Justice Canada.

[B10] Korosec L (2014). Regional variations in self-protection in Canada. Violence Vict.

[B11] Gomez D, Saunders N, Greene B, Santiago R, Ahmed N, Baxter NN (2020). Firearm-related injuries and deaths in Ontario, Canada, 2002-2016: a population-based study. CMAJ.

[B12] Lawson F, Schuurman N, Amram O, Nathens AB (2015). A geospatial analysis of the relationship between neighbourhood socioeconomic status and adult severe injury in Greater Vancouver. Inj Prev.

[B13] Karkada M, Bennett N, Erdogan M, Kureshi N, Tansley G, Green RS (2022). A population-based study on the epidemiology of firearm-related injury in Nova Scotia. Injury.

[B14] Bang F, McFaull S, Cheesman J, Do MT (2019). The rural–urban gap: differences in injury characteristics. Health Promot Chronic Dis Prev Can.

[B15] Catalogue No The Canadian Index of Multiple Deprivation [Internet]. The Canadian Index of Multiple Deprivation [Internet]. Ottawa (ON): Statistics Canada [Catalogue No.

[B16] Newcombe RG, st ed (2012). Confidence intervals for proportions and related measures of effect size. Newcombe RG.

[B17] (2019). B.C.’s health boundaries, Version 2018–B.C. BC Ministry of Health.

[B18] Langmann C (2021). Suicide, firearms, and legislation: a review of the Canadian evidence. Prev Med.

[B19] Navaneelan T, Catalogue No Suicide rates: an overview. Ottawa (ON); Statistics Canada; 2012 [cited 2024 Feb 05]. [Catalogue No.

[B20] Channer NS, Biglieri S, Hartt M, Hartt M, Biglieri S, Rosenberg M, Nelson S Aging in rural Canada. Bristol University Press.

[B21] Moazzami B (2015). Strengthening rural Canada: fewer & older: the population and demographic dilemma in rural British Columbia. Government of Canada.

[B22] Burrows S, Auger N, Gamache P, Hamel D (2013). Leading causes of unintentional injury and suicide mortality in Canadian adults across the urban-rural continuum. Public Health Rep.

[B23] Novilla-Surette EM, Shariff SZ, Le B, Booth RG, Geriatr J (2022). Trends and factors associated with suicide deaths in older adults in Ontario, Canada. Can Geriatr J.

[B24] Casant J, Helbich M (2022). Inequalities of suicide mortality across urban and rural areas: a literature review. Int J Environ Res Public Health.

[B25] Finley CJ—, Hemenway D, Clifton J, Brown DR, Simons RK, Hameed SM (2008). The demographics of significant firearm injury in Canadian trauma centres and the associated predictors of inhospital mortality. Can J Surg.

[B26] Saunders NR, Hepburn CM, Huang A, et al (2021). Firearm injury epidemiology in children and youth in Ontario, Canada: a population-based study. BMJ Open.

[B27] Saunders NR, Lee H, Macpherson A, Guan J, Guttmann A (2017). Risk of firearm injuries among children and youth of immigrant families. CMAJ.

[B28] Saunders NR, Guan J, Macpherson A, Lu H, Guttmann A (2020). Association of immigrant and refugee status with risk factors for exposure to violent assault among youths and young adults in Canada. JAMA Netw Open.

[B29] Langmann C (2020). Effect of firearms legislation on suicide and homicide in Canada from 1981 to 2016. pone.

[B30] Magee LA (2020). Community-level social processes and firearm shooting events: a multilevel analysis. J Urban Health.

[B31] Drake SA, Lemke MK, Yang Y (2022). Exploring the complexity of firearm homicides in Harris County, Texas, from 2009 to 2021: implications for theory and prevention. Soc Sci Med.

[B32] Czechowski K, Sylvestre J, Gogosis E, et al (2022). Cycles of instability: proximal and distal influences on residential instability among people with histories of homelessness in three Canadian cities. J Community Psychol.

[B33] McLellan H (2023). Firearm availability and suicide in Canada: examining the effects of gun control, unemployment, divorce and sex on firearm suicides from 2009-2020 [dissertation]. Memorial University of Newfoundland.

[B34] Yanchar NL, Beno S (2018). Can we do better?: A Canadian perspective on firearm injury prevention. Ann Surg.

[B35] Stamatel JP, Stamatel JP (2021). Gun control. ABC-CLIO.

[B36] Lee J (2021). A comparative analysis of gun policy in Canada and the United States [thesis]. Trinity College.

[B37] Heinmiller BT, Hennigar MA (2022). Aiming to explain: theories of policy change and Canadian gun control. University of Toronto Press.

[B38] Brown RB (2020). The ghost of the long-gun registry: Prime Minister Justin Trudeau and gun control in Canada, 2015-2019. The ghost of the long-gun registry: Prime Minister Justin Trudeau and gun control in Canada, 2015-2019. Can Stud.

[B39] Bennett N, Karkada M, Erdogan M, Green RS (2022). The effect of legislation on firearm-related deaths in Canada: a systematic review. CMAJ Open.

[B40] Patel J, Leach-Kemon K, Curry G, Naghavi M, Sridhar D (2022). Firearm injury—a preventable public health issue. Lancet Public Health.

[B41] Santaella-Tenorio J, Villaveces A, Galea S (2016). What do we know about the association between firearm legislation and firearm-related injuries. Epidemiol Rev.

[B42] Lavoie M, Cardinal L, Chapdelaine A, St-Laurent D (2001). The storage of household long guns: the situation in Quebec. Chronic Dis Can.

[B43] Anglemyer A, Horvath T, Rutherford G (2014). The accessibility of firearms and risk for suicide and homicide victimization among household members: a systematic review and meta-analysis. Ann Intern Med.

[B44] Dahlberg LL, Ikeda RM (2004). Guns in the home and risk of a violent death in the home: findings from a national study. Am J Epidemiol.

[B45] Alphonsus L, Filler R, Strauss R, Silva T, Kobylianski J (2023). Tackling the increasing public health impact of firearms: a call to action [position paper]. Ontario Medical Students Association.

[B46] (2024). National Ambulatory Care Reporting System. Population data BC (PopData).

[B47] Zeoli AM, Malinski R, Brenner H (2020). The intersection of firearms and intimate partner homicide in 15 nations. Trauma Violence Abuse.

[B48] Holliday CN, Kahn G, Thorpe RJ, Shah R, Hameeduddin Z, Decker MR (2020). Racial/ethnic disparities in police reporting for partner violence in the National Crime Victimization Survey and survivor-led interpretation. J Racial Ethn Health Disparities.

[B49] Gezinski LB (2022). “It’s kind of hit and miss with them”: a qualitative investigation of police response to intimate partner violence in a mandatory arrest state. J Fam Violence.

[B50] Heymans MW, Twisk JW (2022). Handling missing data in clinical research. J Clin Epidemiol.

